# Multiple Breast Cancer Cell-Lines Derived from a Single Tumor Differ in Their Molecular Characteristics and Tumorigenic Potential

**DOI:** 10.1371/journal.pone.0055145

**Published:** 2013-01-25

**Authors:** Goar Mosoyan, Chandandeep Nagi, Svetlana Marukian, Avelino Teixeira, Anait Simonian, Lois Resnick-Silverman, Analisa DiFeo, Dean Johnston, Sandra R. Reynolds, Daniel F. Roses, Arevik Mosoian

**Affiliations:** 1 Department of Medicine, Mount Sinai School of Medicine, New York, New York, United States of America; 2 Department of Pathology, Mount Sinai School of Medicine, New York, New York, United States of America; 3 HemoShear, Charlottesville, Virginia, United States of America; 4 Department of Oncological Sciences, Mount Sinai School of Medicine, New York, New York, United States of America; 5 Case Western Reserve University, Department of Case Comprehensive Cancer Center, Cleveland, Ohio, United States of America; 6 Department of Medical Laboratory Sciences, Hunter College, New York, New York, United States of America; 7 Department of Dermatology, New York University School of Medicine, New York, New York, United States of America; 8 Department of Surgery, New York University School of Medicine, New York, New York, United States of America; Florida International University, United States of America

## Abstract

**Background:**

Breast cancer cell lines are widely used tools to investigate breast cancer biology and to develop new therapies. Breast cancer tissue contains molecularly heterogeneous cell populations. Thus, it is important to understand which cell lines best represent the primary tumor and have similarly diverse phenotype. Here, we describe the development of five breast cancer cell lines from a single patient’s breast cancer tissue. We characterize the molecular profiles, tumorigenicity and metastatic ability *in vivo* of all five cell lines and compare their responsiveness to 4-hydroxytamoxifen (4-OHT) treatment.

**Methods:**

Five breast cancer cell lines were derived from a single patient’s primary breast cancer tissue. Expression of different antigens including HER2, estrogen receptor (ER), CK8/18, CD44 and CD24 was determined by flow cytometry, western blotting and immunohistochemistry (IHC). In addition, a Fuorescent *In Situ* Hybridization (FISH) assay for *HER2* gene amplification and p53 genotyping was performed on all cell lines. A xenograft model in nude mice was utilized to assess the tumorigenic and metastatic abilities of the breast cancer cells.

**Results:**

We have isolated, cloned and established five new breast cancer cell lines with different tumorigenicity and metastatic abilities from a single primary breast cancer. Although all the cell lines expressed low levels of ER, their growth was estrogen-independent and all had high-levels of expression of mutated non-functional p53. The *HER2* gene was rearranged in all cell lines. Low doses of 4-OHT induced proliferation of these breast cancer cell lines.

**Conclusions:**

All five breast cancer cell lines have different antigenic expression profiles, tumorigenicity and organ specific metastatic abilities although they derive from a single tumor. None of the studied markers correlated with tumorigenic potential. These new cell lines could serve as a model for detailed genomic and proteomic analyses to identify mechanisms of organ-specific metastasis of breast cancer.

## Introduction

Breast cancer is one of the leading causes of cancer death in women. Breast cancer cell lines have been used widely to study breast cancer biology, to screen new drugs and to identify pathways leading to suppression of cancer growth and metastases. The most commonly used breast cancer cell lines were established decades ago [1], [2], and only a few breast cancer cell lines have been established more recently, mainly due to difficulties in culturing breast cancer cells without surrounding stromal cells.

Breast cancer is recognized to be a molecularly heterogeneous disease. Markers such as estrogen receptor (ER), progesterone receptor (PR) and HER2 are used to make disease prognoses and to select specific therapies. A large percentage of breast cancer tumors express the estrogen receptor alpha (ERα). A common treatment for patients carrying these tumors is the ER antagonist 4-hydroxytamoxifen (4-OHT), but some of these tumors develop resistance to the treatment. There are reports that up-regulation of the HER2 receptor may mediate 4-OHT resistance in ER positive tumors [3]. The p53 tumor suppressor protein is also a critical mediator of the anti-proliferative and pro-apoptotic effects of several treatments used for breast cancer. While there are several reports indicating functional interactions between the ERα and p53 pathways [4], [5], [6], [7], [8], the impact of these interactions during anti-hormone treatments is still unclear.

The aim of this work was to study the correlation of ER, p53, CD44 and CD24 expression with proliferation, tumorigenicity and metastatic potential of breast cancer cells. To this end, we isolated and cloned five human breast cancer cell lines from a single primary breast cancer tumor derived from a single patient. We characterized these cell lines that appeared to differ in their tumorigenic and metastatic potential in immune compromised nude mice. All breast cancer cell lines express low levels of ER and HER2 receptor although their proliferation is not dependent on estrogen. Here we show that low doses of 4-OHT (an estrogen antagonist) induced rather than inhibited proliferation of these breast cancer cells that were ER positive, *HER2* receptor positive and had non-functional p53. In the present work we analyzed the newly developed breast cancer cell lines for their tumorigenicity and metastatic potential in nude mice. These cell lines could serve as an important model for detailed genomic and proteomic analysis to identify mechanisms of organ-specific metastasis of breast cancer.

## Results

### Cloning of Breast Cancer Cell Lines Derived from the Same Tumor

Cell lines from a single primary invasive ductal breast carcinoma of a 35 year old woman were established in tissue culture as detailed in the Methods section. The original tumor was an invasive ductal carcinoma, stage 1, without lymph node metastases (0/15), described as diploid with a high proliferation index. More than 50% of the original tumor cells expressed estrogen receptors and/or progesterone receptors and HER2 in formalin-fixed and paraffin-embedded tissue by IHC. The standard method of assessing HER2 and ER status in breast cancer tissue from patients in 1999 when the tumor was resected was IHC at our institution.

**Figure 1:**
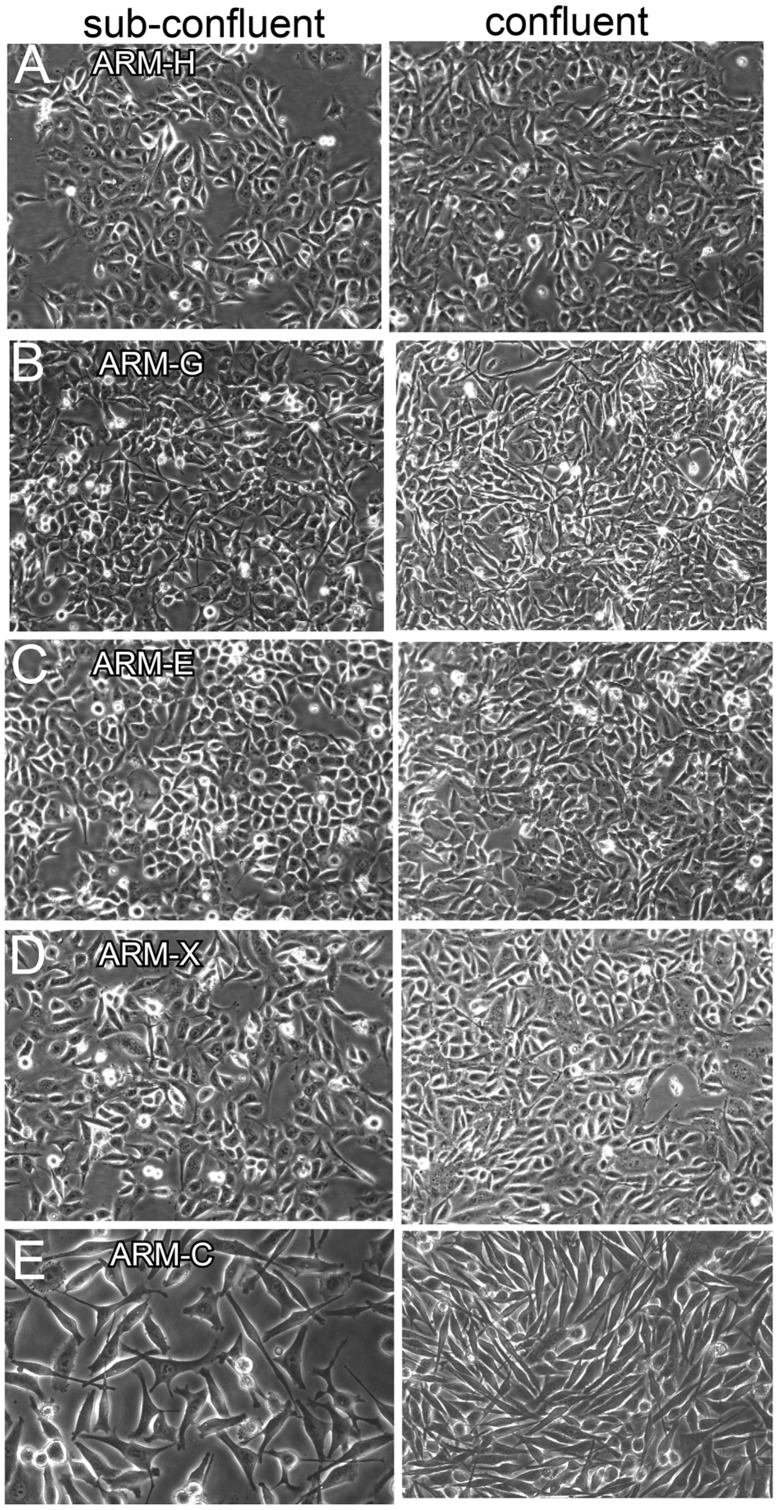
Phase contrast photomicrographs of breast cancer cell lines at sub confluent and confluent stages. (A) ARM-H (B) ARM-G (C) ARM-E (D) ARM-X cell lines (magnification x200).

Clones of several cell lines were produced from the primary breast cancer tissue by limiting dilution and established as separate cultures. Microscopically, all clones had homogenous “plasmacytoid” appearance (Figure 1A, B, C, D, E). Cells were adapted to growth in serum free medium and split once a week at a ratio of 1∶4. All assays were performed with cells maintained in serum-free medium. Each breast cancer cell line was frozen starting from passage five and was passed up to 50 passages in culture. The doubling time for all clones is 24–36 hours.

### Tumorigenicity of Human Breast Cancer Cell Lines

**Figure 2:**
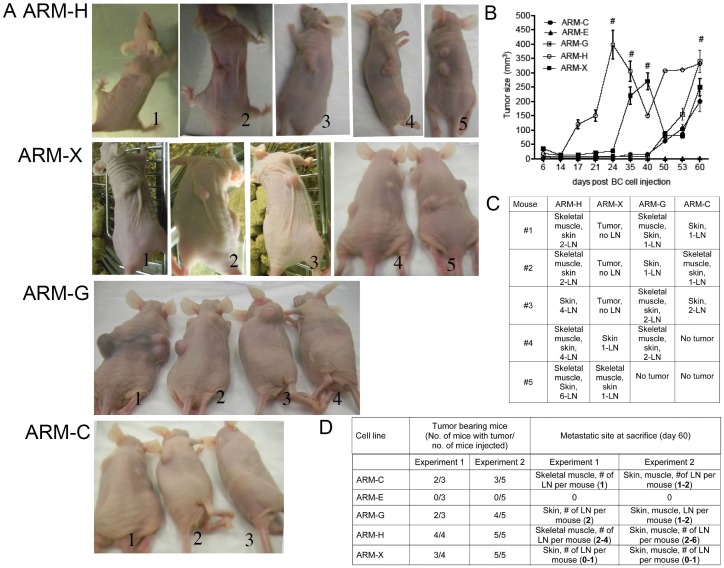
Breast cancer cell lines induce tumors in nude mice and metastasize to different organs. (A) Five female BALB/c nude mice received an injection of different breast cancer cells. #-Time when mice were sacrificed. Day 60 is the end of the experiment, mice were sacrificed and tumors were excised. (B) A comparison between different breast cancer cell lines in tumor growth in nude mice. The mean tumor size in each cell line group was compared. (C) Metastases of breast cancer cell lines in injected nude mice (D) Summary table of tumorigenicity and metastatic potential of breast cancer cell lines.

We evaluated the tumorigenicity of the breast cancer cell lines by injecting 2.5×10^6^ cloned cells per mouse into groups of 8 or 9 nude mice in two separate experiments. We chose to inject a rather high cell number to ensure that clones, which did not produce tumors in nude mice, were truly non-tumorigenic. Five clones were selected for further study because they differed significantly in the tumorigenic potential. ARM-H was particularly tumorigenic, inducing tumors in all mice in both experiments, and ARM-X induced tumors in eight out of nine mice (Figure 2 A, B, C, D). In contrast, the ARM-E cell line did not induce tumors in any recipient mouse in either experiment. The two other clones had intermediate degrees of tumorigenicity, inducing tumors in some but not all mice. In addition we observed a significant difference in the kinetic of tumor development in nude mice by different breast cancer cell lines.

Two mice injected with ARM-H cells developed tumors at day 24 post injection with an average tumor size of 398 mm^3^ (Figure 2A and B). According to the local Institutional Animal Care and Use Committee mice have to be euthanized before the tumor exceeds the maximum allowable size (diameter ∼1 cm). Another 2 mice injected with the same ARM-H cell line were sacrificed at day 35 with tumor size 305 mm^3^ (Figure 2A and B) and the last mouse injected with ARM-H cell line was sacrificed on day 60 with tumor size 332 mm^3^ (Figure 2 A, B).

ARM-X was the only other cell line that developed tumors of critical size in nude mice before 60 days post injection. Two mice injected with ARM-X cell line were sacrificed on day 40 with mean tumor size 270 mm^3^. Three other mice injected with ARM-X cell line were euthanized on day 60 with tumor size 250 mm^3^ (Figure 2A,B).

Two out of three mice injected with cell line ARM-G developed tumors and 4 out of 5 mice developed tumors in the two respective experiments (Figure 2A and B). ARM-G cell injection induced late tumor development, but the tumor size increased dramatically in the last 10 days before mice were sacrificed (day 60) with a mean tumor size 340 mm^3^ (Figure 2A and B).

Two out of three mice injected with cell line ARM-C developed tumors in experiment 1; and 3 out of 5 mice in experiment 2 (Figure 2A, B and C). The tumor size of mice injected with ARM-C cell line on day 60 reached 200 mm^3^ (Figure 2A and B).

All cell lines also induced metastases, however to a different extents and with a different target tissues including, skin, skeletal muscles and lymph nodes. The important difference between the cell lines was the number of enlarged and infiltrated lymph nodes. The cell line ARM-H infiltrated on average 4 lymph nodes in each mouse (Figure 2 C). The other three cell lines infiltrated on average 1 or 2 lymph nodes. Importantly, there was a statistically significant difference in tissue specific metastases of different breast cancer cells. In mice injected with ARM-H cell line metastases were detected in skeletal muscles, skin and lymph nodes in 4 out of 5 mice. In contrast, all five ARM-X injected mice, developed tumors, but only 2 formed skin and lymph node metastases, suggesting that the ARM-X cell line is less metastatic then the ARM-H cell line. These results indicate that multiple clones of human breast cancer cells differed in their ability to induce tumors in nude mice, in the kinetic of tumor formation and metastatic capacity.

### Characterization of Breast Cancer Cell Lines Isolated from Primary Breast Cancer Tissue

**Figure 3:**
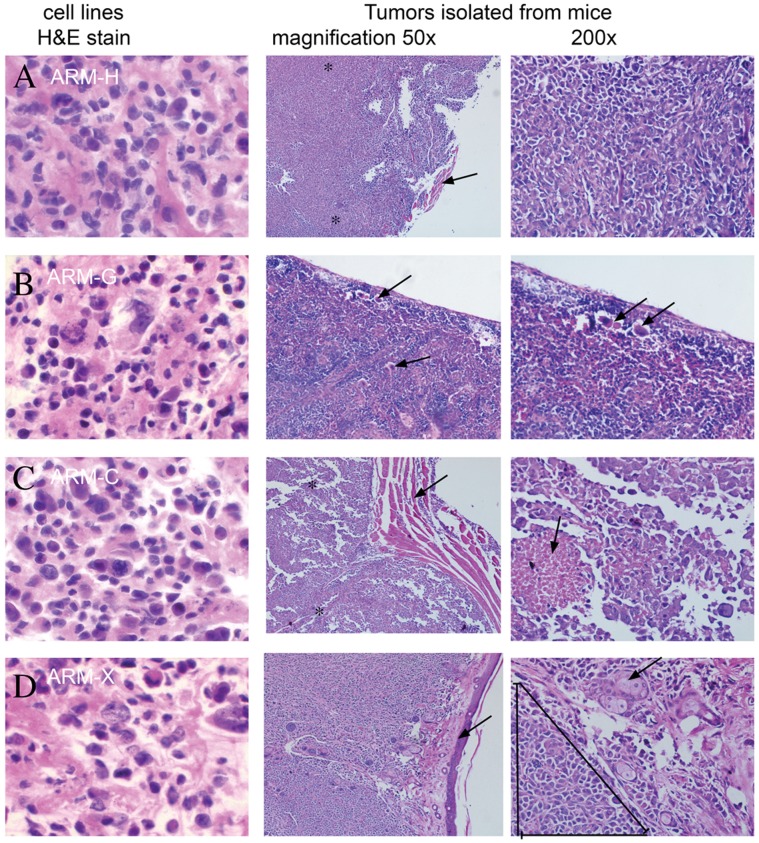
Photomicrographs of H&E stains of human breast cancer cell lines and metastatic tumors from mice. (A) ARM-H (middle, 50×) shows focal residual skeletal muscle (arrow) almost completely replaced and infiltrated by invasive adenocarcinoma (between asterisks); (right, 200×) the same area shows sheets of markedly atypical and pleomorphic carcinoma cells with numerous mitotic figures in a haphazard growth pattern. (B) ARM-G (middle, 50×) lymph node with deposits of metastatic tumor cells within the peripheral sinuses and parenchyma (arrows); (right, 200×) same lymph node with numerous scattered metastatic tumor cells; tumor cells show enlarged nuclei, significant atypia and pleomorphism when compared to the surrounding normal lymphocytes, arrows indicate deposits of tumor cells. (C) ARM-C (middle, 50×) shows sheets of adenocarcnoma cells (between asterisks) invading into adjacent skeletal muscle (arrow); (right, 200×) view of the same area shows markedly pleomorphic and atypical tumor cells with areas of tumor cell necrosis (arrow). (D) ARM-X (middle, 50×) skin (arrow on epidermis), underlying adnexal structures and dermis completely replaced by invasive adenocarcinoma entrapping sweat glands and normal follicles; (right, 200×) the same section showing the markedly atypical tumor cells with mitoses, pleomorphic nuclei, and high nuclear to cytoplasmic ratio invading the dermis and surrounding adnexal structures (triangle); arrow indicates skin adnexal structures.

**Figure 4:**
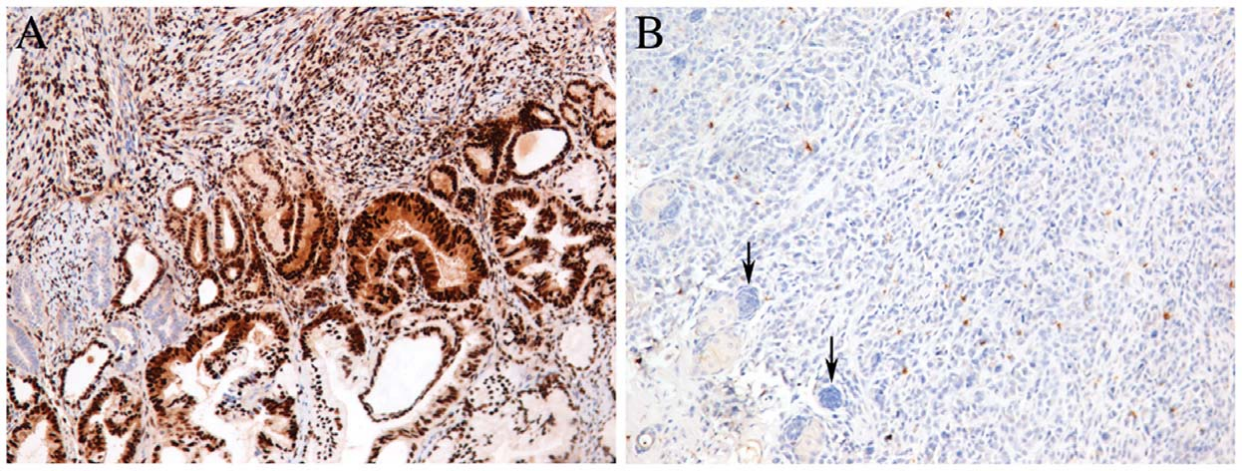
IHC stain of ERα in tumor isolated from nude mice. (A) positive control human breast cancer tissue, (B) tumor from breast cancer cell line ARM-H derived from nude mice, arrows indicate negative mouse nodules.

Histological analysis of breast cancer cells invading skin, muscle and lymph nodes are presented in Figure 3A, B, C, D. IHC of the control tissue from a different patient with breast cancer (used as a positive control) showed conventional nuclear staining of ER (Figure 4A). However, tumor tissue derived from a nude mouse injected with ARM-H cells showed unusually scattered cytoplasmic staining for ER (Figure 4B).

**Figure 5:**
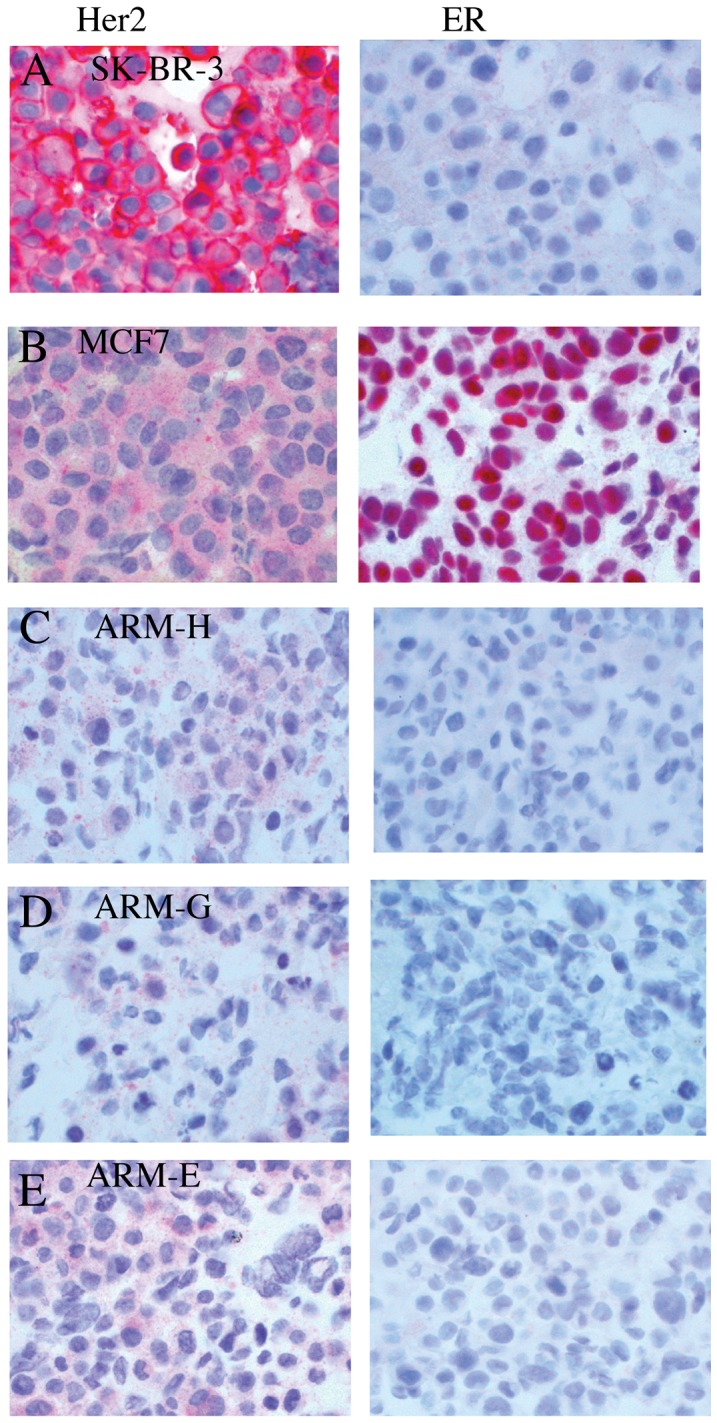
Photomicrographs of IHC stain of ER and HER2 expression and western blotting analyses of ER-expression and response to E2 in breast cancer cell lines. (A) SK-BR-3 (B) MCF-7 (C) ARM-H (D) ARM-G (E) ARM-E cell lines.

All of the following studies were performed with breast cancer cell lines that have only been cultured *in vitro* and never passed in mice. IHC analyses for HER2 and ER in the cultured breast cancer cell lines showed the same unusual phenotype observed in the tumors from nude mice injected with breast cancer cell lines. The control SK-BR-3 breast cancer cell line had classical membrane staining of HER2 (Figure 5A) while our new breast cancer cell lines had more uniform and diffuse cytoplasmic staining (Figure 5C, D, E). As was expected, ER staining was highly positive in MCF-7 cells (Figure 5B), but only faint in the cytoplasm of the newly established breast cancer cell lines (Figure 5C, D, E). All five clones showed low level of HER2 and ER expression by IHC.

In addition, clone ARM-C was positive for carcinoembrionic antigen (CEA) (data not shown).

**Figure 6:**
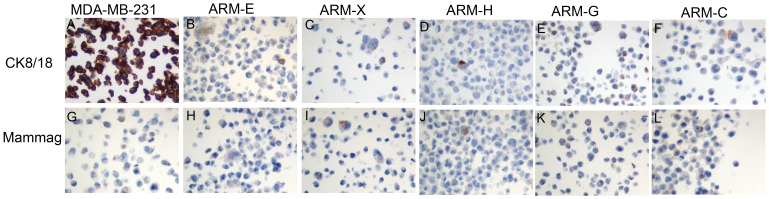
Photomicrographs of IHC stain of CK8/18 and mammaglobin for breast cancer cell lines. (A) MDA-MB-231, (B) ARM-E, (C) ARM-X, (D) ARM-H, (E) ARM-G, (F) AMR-C; mammaglobin G) MDA-MB-231, (H) ARM-E, (I) ARM-X, (J) ARM-H, (K) ARM-G, (L) AMR-C (magnification 400×).

To further characterize the molecular subtype of these breast cancer cell lines and to prove their epithelial origin, we analyzed expression of cytokeratins (CKs). The control MDA-MB-231 breast cancer cell line was strongly positive for CK8/18 (Figure 5A). All five breast cancer cell lines are positive for CK8/18 to different extents, suggesting their luminal origin; cell lines ARM-G and ARM-C (60–65% respectively) had the highest expression (Figure 6 B, C, D, E, F) [2], [9], [10], [11]. All five-breast cancer cell lines were negative for CK5/6 (data not shown) but expressed another breast specific antigen mammaglobin (Figure 6 H, I, J, K, L).

The summary of the tumorigenicity of the breast cancer cell lines in nude mice and the percentage of cells staining positive for the different antigens are shown in Table 1. We did not observe any correlation between the expression of any of the studied markers with the tumorigenicity.

**Table 1:** Summary of the results for tumorigenicity and percentage of expression of different antigens in breast cancer cell lines.

Cell lines	MCF7	MDA-MB-231	ARM-C	ARM-E	ARM-G	ARM-H	ARM-X
Tumorigenicity, twoexperiments (%)	n/d	n/d	62% (5/8)	0	75% (6/8)	100% (9/9)	88%(8/9)
CK8/18 (%)	100%	100%	25%	5%	35%	5%	10%
Mammaglobin (%)	1%	60%	65%	10%	60%	1%	5%
ER (%)	100%	n/d	5%*	10%*	15%*	5%*	5%*
Her2 (%)	1%	n/d	10%	5%	5%	15%	10%

* Indicates scattered cytoplasmic staining.

**Figure 7:**
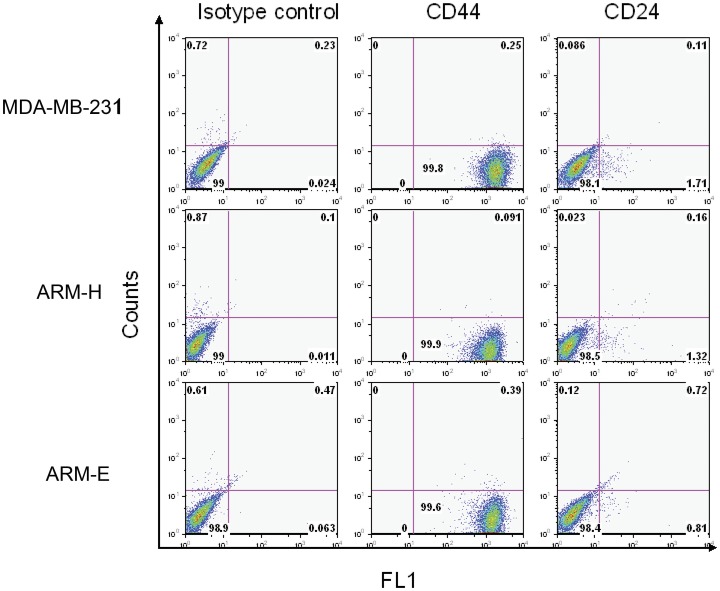
Expression of CD44/CD24 receptors on breast cancer cells. The plots depict CD44, CD24 and an isotype control antibody staining of ARM-H, ARM-E and control MDA-MB-231. This is one representative experiment of three independent experiments.

We also examined cell surface receptors CD44/CD24, in the most tumorigenic (ARM-H) and the least tumorigenic (ARM-E) of the five cell lines. Using FACS analysis, both cell lines expressed the CD44^high^/CD24^low^ phenotype and there were no quantitative differences in the levels of the two receptors expressed by either cell line (Figure 7). Nor did the expression level differ from the positive control breast cancer cell line MDA-MB-231 (Figure 7).

**Figure 8:**
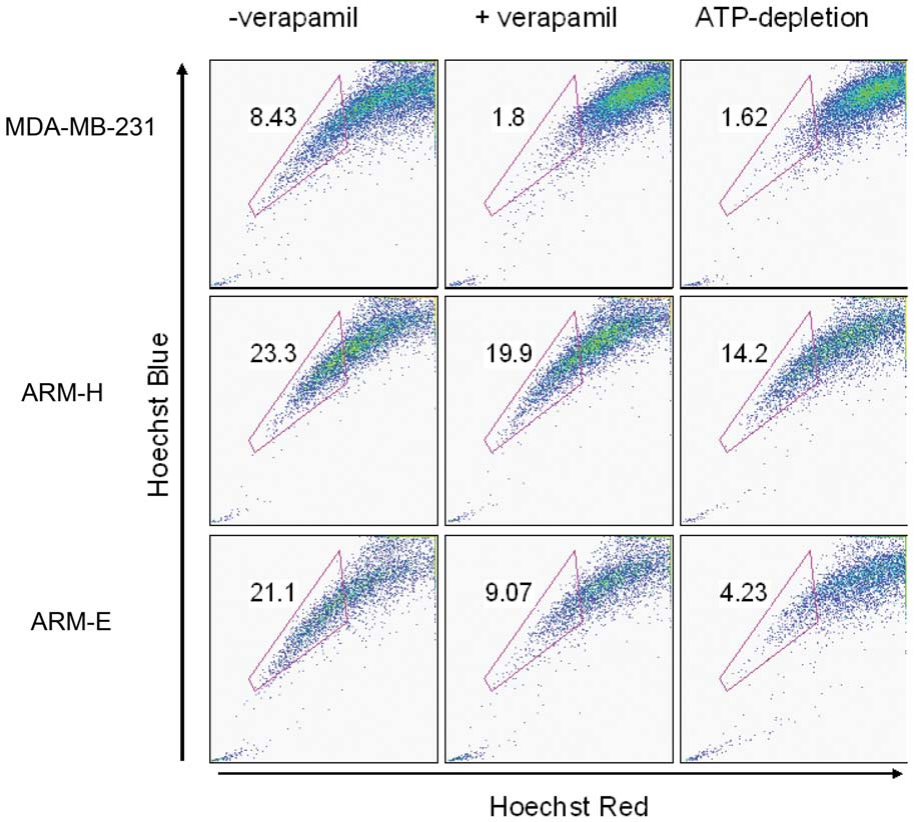
Side population profile of breast cancer cell lines ARM-H, ARM-E and control MDA-MB-231. The side-population cells are indicated in enclosed boxes and the percentage of the cells in this region is indicated in each panel. These data are representative of two independent experiments.

Another proposed marker of tumorigenicity is “side population” (SP) characteristic. The population of cancer cells that efflux chemotherapy drugs and therefore account for resistance of cancer to chemotherapy is identified as side population [12]. We examined SP in the most tumorigenic clone (ARM-H), a non-tumorigenic clone (ARM-E) and as a control we used a well-known, highly tumorigenic breast cancer cell line, MDA-MB-231. All three cell lines contained SP characteristics as shown by incubation with Hoechst dye in the presence and absence of blockers of dye exclusion (Figure 8). Verapamil and ATP depletion (using sodium azide and deoxyglucose) block the dye exclusion mechanism by inhibiting the function of the P-glycoprotein transporter and the other ABC transporters respectively [13]. However we did not perform sorting of the side population from newly established breast cancer cell lines to determine the direct correlation of SP with tumorigenicity since the level of SP in tumorigenic (ARM-H) and –non-tumorigenic (ARM-E) cell lines was comparable (23.3%–21.1%).

### *HER2 Gene* Amplification

Amplification of the *HER2* gene has been shown to be both a prognostic and predictive marker in the outcome of breast cancer disease [14], [15]. To determine whether the *HER2* gene was amplified in the newly established breast cancer cell lines, we used FISH. (Because primary breast cancer tissue was no longer available, the assay was not performed on original patient breast cancer tissue). The *HER2* gene which is localized on chromosome 17 region 17q11.2-12 was labeled red and the centromere of chromosome 17 labeled green. A normal cell exhibits two signals for each color and a ratio of *HER2* to Centromere 17 is normally 1.0. If there is *HER2* gene amplification, multiple copies of *HER2* signals are observed.

**Figure 9:**
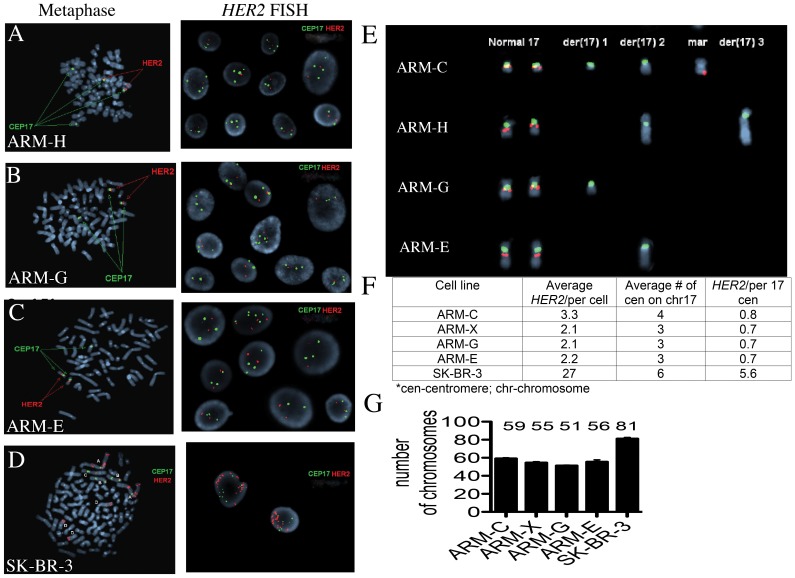
Representative metaphase (left) and interface (right) from four-breast cancer cell lines. Each cell nucleus contains *HER2* signals (red) and centromere 17 signals (green). (A) ARM-H (B) ARM-G (C) ARM-E and (D) SK-BR-3; (E) composite karyotype of chromosome 17 and derivatives in four cell lines; (F) summary table of FISH analyses; (G) number of chromosomes in breast cancer cell lines.

The amplification ratio of all our breast cancer cell lines ranged from 0.7–0.8. A ratio of less than 1 means that there were more Centromere17 signals than *HER2* signals (Figure 9A, B, C, F). As was expected, the control breast cancer cell line SK-BR had *HER2* amplification with a ratio of 5.6 which is consistent with *HER2* gene amplification reported for this cell line (Figure 9D, F) [16], [17]. All cancer cells have two normal *HER2* signals on chromosome 17. The average numbers of *HER2* signals per cell for cell lines ARM-C, ARM-X, ARM-G and ARM-E were 3.3, 2.1, 2.1, and 2.2 respectively (Figure 9A, B, C, F). However, metaphase FISH from cell line ARM-H revealed two additional centromere 17 signals that lacked the *HER2* gene (Figure 9 A, E). Metaphase FISH from cell lines ARM-G and ARM-E also revealed one additional centromere 17 signal that lacked the *HER2* gene (Figure 9B, C, E, F). The number of chromosomes in each cell line was distinct and varied from 51 for cell line ARM-G to 59 for cell line ARM-C (Figure 9G).

### Aberrant p53 Tumor Suppressor Gene Expression and Function

**Figure 10:**
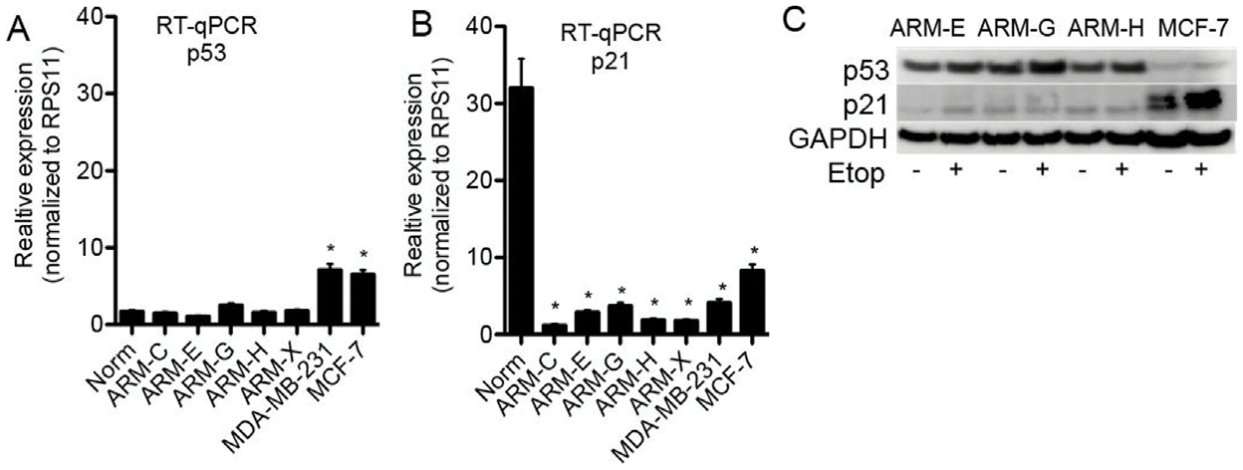
mRNA expression level and western blotting analyses of p53 and p21. RNA was isolated from breast cancer cell lines, converted into cDNA, followed by q-PCR with (A) p53 or (B) p21 specific primers. Relative mRNA expression was calculated after normalization to the ribosomal protein (RPS11) control gene. Each experiment was done at least three times. Data represent mean ± SD. *ANOVA one-way statistical analyses* was used to compare results *p<0.05 considered statistically significant; (C) western blotting analyses of breast cancer cells treated with 5 uM of etoposide for 24 h followed by protein extraction, gel electrophoresis and antibody staining for indicated proteins.

It has been proposed that a CD44^high^ phenotype correlates with over-expression of mutated p53 in tumor tissue [18], [19], suggesting that inactivated p53 expression can aid the survival of immortalized, premalignant cells and may also correlate with the level of *in vivo* tumorigenicity of breast cancer cells [19], [20]. To test the likelihood of this correlation, we measured p53 mRNA expression by real-time PCR in our five breast cancer cell lines, and as a control we used normal breast epithelial cells and two other breast cancer cell lines. Expression of p53 mRNA was up-regulated in the tumorigenic MDA-MB-231, as well as in the non-tumorigenic MCF-7 compared to normal breast epithelium (Figure 10A). However, in the cell lines isolated from the patient's breast cancer tissue, mRNA levels were close to normal regardless of their tumorigenicity status (Figure 10A). By RT-qPCR, we also showed lower expression of p21 mRNA in all newly established breast cancer cell lines regardless of tumorigenicity status compared to normal breast epithelial cells isolated from the same patient donor (Figure 10B).

Next we studied whether p53 is functional. In the presence of functional p53, treatment of cells with the DNA-damaging agent etoposide would up-regulate p21 protein. As a positive control for functional p53 we used the MCF-7 breast cancer cell line. As expected in the MCF-7 cell’s p21 protein level was up-regulated in response to etoposide-treatment (Figure 10C). By contrast, even though high levels of p53 protein appear to be present in our newly derived cell lines, etoposide treatment failed to induce p21 protein-expression (Figure 10C), suggesting that p53 is present at low levels and non-functional in the cells lines derived from the patient's tumor.

Mutational analyses of the *TP53* tumor-suppressor gene revealed the presence of two single nucleotide alterations. In exon three of all five cell lines, there was a homozygous single nucleotide alteration (ID# rs1042522) where cytosine in position 215 was replaced by guanine (C215G), which leads to a change of amino acid proline in position 72 to arginine (P72R) [21]. There was also another heterozygous single nucleotide alteration detected in all five cell lines. In exon seven of the *TP53* gene, guanine at position 797 was substituted by adenine (G797A) leading to a change of amino acid glycine at position 266 to glutamic acid (G266E). These alterations in the *TP53* gene lead to changes in amino acid sequence of the p53 protein and together with functional assay data indicate presence of mutated, non-functional p53 gene in the patient's breast cancer cell lines.

### Effect of Tamoxifen on Growth of Breast Cancer Cell Lines

Estrogen receptor (ER) belongs to a family of nuclear hormone receptors. It plays a key role in regulating growth, differentiation, and tumorigenesis of breast cancer cells when activated by its ligand estrogen. There are two known isoforms of estrogen receptor: ERα and ERβ. We used antibodies to determine ERα expression in breast cancer cell lines and tumors derived from nude mice injected with our cell lines.

**Figure 11:**
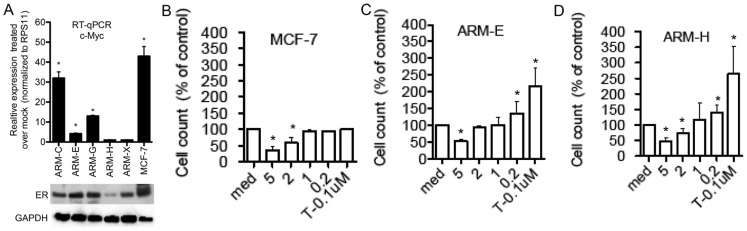
Breast cancer cell lines express different levels of estrogen receptor and respond differently to estrogen and 4-OHT treatments. (A) bottom. Western blotting analyses of breast cancer cells for ER expression and top RT-qPCR for cMYC gene expression in RNA extracted from indicated cells treated with 10 nM of E2 for 24 h. (B) Cytotoxicity assay of MCF-7, (C) ARM-E and (D) ARM-H breast cancer cells treated for 3 days with different concentrations of 4-OHT. Left axis represents the relative percentage of live cells treated with 4-OHT compared to medium-treated cells. Similar results were obtained in three independent experiments. Data represents mean ± SD. Student’s t test was used to compare means of treated versus untreated samples with *p<0.05 considered statistically significant.

Cell lines ARM-C, ARM-E ARM-G and ARM-X expressed nearly similar levels of ERα, only the most tumorigenic cell line (ARM-H) showed less expression of ERα by western (Figure 11A). Next we tested whether estradiol (E2) could stimulate the expression of the ER target gene c-Myc. We tested relative level of c-Myc expression by RT-qPCR after treatment of with 10 nM of E2 for 24 h. As was expected, the MCF-7 cell line responded to E2 treatment by robust up-regulation of c-Myc gene expression (Figure 11A) E2 treatment of cell lines ARM-C, ARM-E and ARM-G triggered moderate expression of c-Myc, whereas cell lines ARM-H and ARM-X failed to activate c-Myc gene expression (Figure 11A).

We next asked whether the ERα antagonist tamoxifen (a common treatment used for breast cancer patients with ERα-positive tumors) had any effect on proliferation of these cell lines. As a positive control we used the MCF-7 breast cancer cell line known to be tamoxifen-sensitive. As was expected, growth of MCF-7 cells was inhibited by high concentrations of tamoxifen (Figure 11B). Interestingly, both non-tumorigenic and tumorigenic ARM breast cancer cell line proliferation was suppressed by high concentrations of tamoxifen. Strikingly, low levels of tamoxifen (100 nM-200 nM) actually induced growth of the newly established breast cancer cells while they did not have any effect on proliferation of MCF-7 cell line (Figure 11C and D).

## Discussion

It is well known that there is great heterogeneity in the antigenic and tumorigenic properties of individual tumor cells within primary lesions of cancer, and there is an ongoing effort to identify individual characteristics of the cells that correlate with their ability to be tumorigenic xenograft models such as immunodeficient *nude* mice. In this study, we minimized confounding factors by isolating and cloning five cell lines of human breast cancer cells derived from a single primary breast cancer tumor from one patient. Our study confirmed the high level heterogeneity in the pattern of antigen expression and tumorigenicity in the different clones that were all derived from the same primary breast cancer tumor. In human breast cancer several characteristics have been suggested as markers of tumorigenicity. These include, “side population characteristics” profile (SP), high expression of CD44 along with low expression of CD24 [22], high expression of HER2 [23] and inactivation and/or high level of expression of p53 [18], [19], [24] with low levels of p21 [25]. In studies designed to determine side population profile it is known that Verapamil blocks the P-glycoprotein transporter and sodium azide in combination of deoxyglucose blocks the dye exclusion mechanism by inhibiting the function of the other ABC transporters including breast cancer resistance protein (BCRP; ABCG2) respectively [13]. In our experiments MDA-MB-231 cell line treatment with verapamil completely blocks die exclusion suggesting that these cells express P-glycoprotein transporter. However in both newly derived breast cancer cell lines ARM-H and ARM-E verapamil only partially blocks die exclusion suggesting the presence of other ATP dependent transporters in addition to P-glycoprotein transporter. Moreover ATP depletion of the non tumorigenic cell line ARM-E significantly blocks die exclusion (57%) however in the most tumorigenic cell line ARM-H ATP depletion only partially (28%) blocks die exclusion suggesting the presence of other types of transporters.

Importantly, in our hands there were no correlations between any of the above-mentioned characteristics and the tumorigenic potential in our newly established breast cancer cell lines.

We could furthermore demonstrate different organ specific metastatic ability of breast cancer cell lines. However, more studies are required, including characterization of expression of different chemokine receptors by breast cancer cell lines, to understand the mechanisms of organ-specific metastatic ability of cancer cell lines.

To test whether the *HER2* gene was amplified in the patient's cancer cell lines, we used the FDA approved clinical -FISH test. The *HER2* gene was not amplified in all five breast cancer cell lines, however it was rearranged. This rearrangement may possibly lead to the unusual scattered cytoplasmic staining of the HER2 protein.

The loss of function of tumor-suppressor gene *TP53* is the most common abnormality in a number of human cancers. Our cell lines showed homozygous (P72R) and heterozygous (G266E) single nucleotide alterations in all five breast cancer cell lines. The single nucleotide alteration of p53 at codon 72 is common and unique to humans. Reports of diminished Mdm2-mediated degradation of altered p53 codon 72R (arginine) compared with p53-72P (proline), [26] correlates with our data that p53 protein was over-expressed in our cell lines. In addition, all cell lines carry another single nucleotide alteration (G797A) leading to replacement of glycine at position 266 to glutamic acid (G266E). This single nucleotide alteration is located in the DNA binding domain of the protein and could most likely be responsible for disrupting normal p53 protein function. Concurrent with this result, we found an inability of p53 to induce p21 protein in response to DNA damaging agent in all five-breast cancer cell lines. We also showed no induction of total p53 protein in response to etoposide treatment in the MCF-7 cell line, which is in agreement with published results on up-regulation of only phosphorylated p53 protein in response to etoposide treatment [27].

Single nucleotide alteration of *TP53* (ID# rs1042522, P72R) has been correlated with reduced disease free and overall survival in women with breast cancer [28]. *TP53* codon *P72R* has even been suggested to be used as a marker to screen individuals at a high risk for breast and pancreatic cancer [28], [29], [30]. This single nucleotide alteration has also been associated with several other cancers including glioma, prostate, non-small cell lung cancer [31], [32], [33], [34], and childhood acute lymphoblastic leukemia [35]. Single nucleotide alteration of the *TP53* gene in the DNA binding domain of exon seven G797A (G266E) has been documented for MDA-MB-435 lines. However, another single nucleotide alteration that we observed (ID# rs1042522, P72R) has not been reported for any of the 41 studied breast cancer cell lines [36], [37].

There are reports of positive therapeutic responses to 4-OHT treatment in patients with ER-positive breast cancer who carry wild-type p53 [38]. We used the patient's ER positive cell lines that have mutated p53 to study their response to 4-OHT. The growth of tumorigenic ARM-H cell line (with low expression of ER and not responding to E2 treatment by target gene c-Myc stimulation) as well as non-tumorigenic cell lines ARM-E (with high expression of ER and responding to E2 treatment by c-Myc gene induction) was suppressed in response to high doses (5–2 µM) but enhanced at low doses (0.1–0.2 µM) of 4-OHT treatment.

It has been reported that the mechanisms of tamoxifen cytotoxicity differ in ER-positive and ER-negative breast cancer cells [39]. Inhibition of proliferation in breast cancer cell line ARM-E by high concentration of 4-OHT could be due to the classical ER antagonist pathway accompanied by changes in cell cycle kinetic parameters. However in cell line ARM-H inhibition of cell proliferation by high doses of 4-OHT could be a result of 4-OHT induced defects in nuclear division and accumulation of cells in S phase in agreement with other published data [39].

Stimulation of cell proliferation in response to low doses of 4-OHT is an interesting observation, which could be due to the presence of non-functional p53 failure to regulate p21 in ARM-E cell line carrying functional ER. Our data are in agreement with recent publications showing (non-functional) p53-status dependent breast cancer cell proliferation after treatment with 4-OHT [5], [38]. However breast cancer cell lines used in these previous studies were all estrogen-dependent in contrast to our cell lines that are estrogen-independent. The authors proposed that loss of p53 function might increase the cross-talk between the ER and the EGFR/HER2 pathways, thus contributing to a proliferative effect of 4-OHT. The mechanism of stimulation of proliferation in response to 4-OHT could be different in ARM-H cell line carrying non-functional ER. A growing number of reports regarding effects of E2 cannot be explained by the classical model of E2 action, which involves the binding to ERs and the interaction of the E2-ER complex with specific DNA sequences linked to E2 target genes. There are several reports from different groups reporting proliferation in response to tamoxifen in ER-negative cells, which is mediated by G protein-coupled receptor GPR30/GPER-1 (GPER-1) [40], [41], [42]. Our cell line with low expression level of ER (ARM-H) showed increased proliferation in response to 4-OHT, possibly suggesting a recruitment of G protein-coupled receptor GPR30/GPER-1 (GPER-1). However, more experiments (including silencing of GPR30/GPER-1 with RNAi) are required to determine the exact mechanism of ER negative breast cancer cell proliferation in response to 4-OHT.

Contrary to current thinking, binding of 4-OHT to ER in breast cancer cells could induce rather than inhibit cancer cell proliferation in settings where the p53 protein is mutated and is present along with amplified or mutated HER2/neu receptors. This includes cases when ER receptors are present but tumor growth is not estrogen dependent. Our findings and reports from other groups suggest that more studies are required to further address the relevance of mutated p53 and HER2/neu in the regulation of the ERα pathway.

In the present work we performed an extensive characterization of the newly developed ARM breast cancer cell lines. Given their different metastatic potential, they could serve as a good model for detailed genomic and proteomic analysis to identify mechanisms of organ-specific metastasis of breast cancer.

To study interactions of human cancer tissues with the host environment, an *in vivo* nude mice system has become a valuable tool in breast cancer research. However, the most widely studied metastatic models of xenografts from established breast cancer cell lines have been developed by several in-vivo passages [43]. Passaging cancer cells in nude mice will alter their phenotype by exposing them not only to mouse mammary tumor virus but to other mouse pathogens as well. This could potentially alter not only the metastatic capacity but also the antigenic profile of cells to the level that *in vivo*-passaged human breast cancer cells fail to represent true human breast cancers. In contrast, these five newly developed breast cancer cell lines were never passaged in mice and display a very different tumorigenic and metastatic potential. Thus these breast cancer cell line xenografts can provide valuable tools to study various important interactions between the tumor and host tissues, including endocrinologic, immunologic, and tumor-stroma interactions.

## Materials and Methods

### Reagents, Hormones, and Antibodies

17-Estradiol (E2), 4-hydroxy-tamoxifen (OHT) and all other reagents were purchased from Sigma (St. Louis, MO) unless otherwise indicated. Antibodies used: *HER2/neu* (Clone L87), carcinoembrionic antigen (CEA) (clone COL-1), epithelial membrane antigen (EMA) (clone VU 4H5), Epithelial Specific Antigen (ESA) (clone VU-1D9) (all from NeoMarkers, Fremont, CA), anti-p21 monoclonal antibody clone SXM30 (BD Pharmingen), anti-Glyceraldehyde-3-Phosphate Dehydrogenase, (GAPDH) clone 6C5 Antibodies (Millipore, Temecula, CA).

### Isolation and Cloning of Human Breast Cancer Cell Lines

This study was conducted using adherence to Helsinki Declaration guidelines. Collection of breast cancer tissue was approved by the New York University School of Medicine Institutional Review Board and was done in accordance with the ethical guidelines for use of human specimens.

The tissues were rinsed in HBSS containing 100 µg/mL gentamicin, 200 U/mL penicillin, 200 µg/mL streptomycin (all from Invitrogen, Carlsbad, CA), dissected into small pieces and digested for 1 h at 37°C with an enzyme mixture comprised of 200 µg/mL collagenase type III (Worthington Biochemical Corporation, Lakewood, NJ) and 1 mg/mL dispase (Boehringer Mannheim, Indianapolis, MN) in HBSS. The digested cells were washed X 3 with 1∶1 dilution of 15 ml HBSS and Ham’s F12 medium (Mediatech, Inc, Herndon, VA); then suspended in Ham’s F12 medium supplemented with 10% FBS, 200 U/mL penicillin, 200 µg/mL streptomycin, and 2 mM L-glutamine. The cells were plated in 6-well tissue culture plates that were coated with collagen type I (Collaborative Biomedical, Bedford, MA). Once cells grew continuously, the concentration of FBS was gradually reduced step-wise every 4 weeks until the cells were fully adapted to long-term growth in serum-free F-12 medium supplemented with 5 µg/mL transferrin (Sigma, St. Louis, MO), 5 µg/mL insulin (Novo Nordisk, Princeton, NJ), 1 µg/mL hydrocortisone, 50 ng/mL triiodothyronine, 20 ng/mL β-estradiol and 30 ng/mL progesterone (all from Sigma).

### Fluorescent *In Situ* Hybridization (FISH) for *HER2* Gene Amplification and Determination of Number of Chromosomes in Breast Cancer Cell Lines

Interphase and metaphase cells were obtained from cultures using standard cytogenetic methods. Fluorescence in situ hybridization (FISH) analysis was performed as previously described with pepsin modification treatment (100 uL 10% pepsin and 2 mL 1% HCl) for five minutes [44]. Codenaturated DNA from cells was hybridized with FISH probes using two DNA probes, *HER2* (17q11.2-12) and CEP17 (17p11.1-q11.1 Alpha Satellite DNA) in Thermobrite (Abbott Molecular, Des Plaines, IL) for three minutes at 73°C and hybridized at 37°C overnight. This is an FDA approved clinical test. A total of 50 nuclei were scored in each of the 6 cell lines and the *HER2* gene amplification was calculated as per manufacturers instructions. To determine the number of chromosomes, five metaphase cells were randomly chosen from each cell line and the number of chromosomes was enumerated.

### Tumorigenicity Assay

The animal protocol was approved by the Institutional Animal Care and Use Committee of New York University School of Medicine and Mount Sinai School of Medicine in compliance with internationally recognized animal guidelines. 2.5×10^6^ cells of each line suspended in 150 µL of phosphate-buffered saline (PBS) were injected subcutaneously into the flanks of 4-week-old athymic female Nu/nu mice (Charles River Laboratories, Wilmington, MA) or from Taconic Farms Inc, NY, without implanting human estrogen patches. The animals were maintained for four to six weeks in 1^st^ experiment and eight weeks in 2^nd^ experiment. Animals were palpated weekly for tumor appearance. Tumor volumes were calculated with the following formula: tumor volume (mm^3^) = 0.5×length (mm)×width^2^ (square mm). When tumor nodules reached 0.5–2 cm in size, mice were euthanized by exposure to CO_2_, tumors were excised and histology was performed at the NYU and Mount Sinai Pathology Core Facility in New York City.

### Detection of Side Population

Side population (SP) characteristics were analyzed based on the ability of the cells to exclude Hoechst 33342 dye [13]. Briefly, cells were re-suspended at 1×10^6^/mL in pre-warmed F12 medium with 2% FCS, 5 µg/mL Hoechst 33342 dye and with or without 50 µg/mL verapamile or 50 µg/mL deoxyglucose (Sigma) and incubated at 37°C for 90 min.

### Western Blot Analyses

For immunophenotyping, cell lysate was electrophoresed and the proteins transferred to PVDF-membrane then probed with monoclonal antibodies directed to breast cancer associated antigens. Strips of membrane were then incubated with monoclonal antibody against HER2/neu (Clone L87), carcinoembrionic antigen (CEA) (clone COL-1), or epithelial membrane antigen (EMA) (clone VU 4H5) (all from NeoMarkers, Fremont, CA) according to manufacturer’s protocol. The membranes were washed in TBS (Sigma) containing 0.1% tween 20 followed by incubation with HRP- labeled secondary antibodies (Sigma) according to manufacturer’s instructions. Proteins were visualized using enhanced chemiluminescent ECL substrate (Amersham Pharmacia, Piscataway, NJ) as per the manufacturer’s instructions.

For the etoposide assay, protein extracts were prepared by lysing cells with RIPA- buffer (Pierce) containing proteases (Pierce). Equal amounts of total proteins were separated on a 4–10% SDS-PAGE gel (Invitrogen) and transferred to a PVDF-membrane (Invitrogen). Anti-p21 monoclonal antibody clone SXM30 (BD Pharmingen). Anti-Glyceraldehyde-3-Phosphate Dehydrogenase, (GAPDH) clone 6C5 Antibodies (Millipore,Temecula, CA). Protein signals were revealed using AmershamTM ECL western-blotting Detection Reagents and AmershamTM Hyperfilm ECL. Anti-HER2/neu Ab-20 clone L87+2ERB19 and anti-P53 (clone DO-7+BP-12) (Thermo scientific, Fremont, CA). Each experiment was repeated at least twice.

### *TP53* Gene Mutation Screening

Cells were grown to 70–80% confluence, total genomic DNA was isolated using the QIAamp DNA Blood mini Kit (Qiagen). Genomic DNA isolated from 5 different breast cancer cell lines was used to analyze the coding region of human *TP53* gene for mutation analysis by sequencing (*TP53*; NCBI GeneID: 7157; NM_001126112.1; CCDS 11118.1, Builds 35.1–37.1), with sequence analysis across the coding ten exons including 10 bp 5′ and 3′ intron sequence, covered by up to 13 amplicons (average 400 bp per amplicon). PCR reaction was cleaned-up, double strand sequencing with PCR primers was used and data was analyzed based on variants: NM_001126112.1, NM_000546.4, NM_001126113.1 and NM_001126114.1, NM_00126115.1, NM_00126116.1 and NM_00126117.1 by Genewiz (South Plainfield, NJ).

### Flow Cytometry

Expression of CD44 and CD24 receptors was analyzed by FACS. The cells were harvested with 0.05% trypsin (Invitrogen), washed and incubated with anti-human CD44 (Sigma, clone c7923) or anti-human CD24 antibodies (Becton Dickenson Biosciences, San Jose, CA) or isotype control antibodies (mouse immunoglobulin) (Sigma). Cells were stained similarly for expression of HER2/neu (Clone 9G6.10), CEA (clone COL-1), and Epithelial Specific Antigen (ESA) (clone VU-1D9) (all from NeoMarkers, Fremont, CA) and for expression of NKI/C3 (Monosan, Netherlands). Fluorescence was detected using a FACScan (BD Immunocytometry Systems, San Jose, CA) and analyzed using FlowJo software (Tree Star, Beckman Coulter, Fullerton, CA).

### Hematoxylin and Eosin (H&E) Staining

Breast cancer cell line clots were prepared by the addition of equal parts of thrombin and fibrin (expired platelets from the blood bank) followed by resuspension and cytocentrifugation to a form cell pellet. The clots were fixed in 10% neutral buffered formalin, processed, sectioned and stained with H and E using standard protocols.

### Immunocytochemistry (IHC)

AgarCyto cell blocks were prepared based on the method of Kerstens et al., [45] using agar as an intermediate embedding medium. Breast cancer cells were briefly fixed in 10% neutral buffered formalin and resuspended in 0.3 mL of 2% liquid agarose at 65°C. The solidified agar–cell pellet was then embedded in paraffin. Slides were cut at 4 microns, baked at 90°C and stained according to manufacturers' recommendations. Detection antibodies included mammagloblin clone 31A5 (Cell Marque), CK 8/18, clones B22.1 and B23.1, CK 5/6 clones D5, 16B4 (Ventana Medical Systems) and HER2/neu Clone L87 (NeoMarkers, Fremont, CA). Visualization was done using the Ventana iView DAB Detection kit followed by counter-staining with Harris Hematoxylin.

Immunostaining for CK8/18, mammaglobin, ER and Her2 antigens for each cell line was evaluated for both staining intensity and percentage of positive epithelial cells in five randomly selected areas.

### Real-time PCR

RNA was isolated from 1×10^7^ cells using 1 mL of TRIzol (Invitrogen) followed by DNase I treatment (Qiagen) according to manufacturer’s instructions. cDNA synthesis was done by using oligo-dT primers and an Omniscript reverse transcription kit (Qiagen). PCR primer sequences for p53, p21 and ribosomal protein S11 (RPS11) were as follows (forward and reverse, respectively): p53-(5′ CCGCAGTCAGATCCTAGCG-3′ and 5′-AATCATCCATTGCTTGGGACG-3′); p21 (5′-ACTCTCAGGGTCGAAAACGG-3′ and 5′-CCTCGCGCTTCCAGGACTG-3′). RPS11 (5′-GCCGAGACTATCTGCACTAC-3′ and 5′-ATGTCCAGCCTCAGAACTTC-3′). Quantification of gene expression was based on the threshold cycle (C_T_-value), defined as the first cycle number with detectable fluorescence above background. Relative quantification of specific gene expression was calculated by comparing C_T_-values of individual genes after normalizing to a reference - gene ribosomal protein. Real time PCR for mRNA expression profiles were performed on total RNA isolated from cells. Primers and kits for preparation of cDNA were from Qiagen company (Valencia, CA). All mRNA transcripts were measured by RT-qPCR where fold change in mRNA expression of breast cancer cells was calculated as compared with normal breast epithelial cells after normalization to the ribosomal protein (RPS11) control gene. Each experiment was done at least three times. Data represent mean ± SD. Student’s *t* -test was used to compare means of treated versus control samples. P<0.05 was considered to be statistically significant.

### Cell Proliferation Assays in Response to Different Treatments

17β-Estradiol (E2), was purchased from Sigma (St. Louis, MO) unless otherwise indicated. The effect of 17β-estradiol (E2) and 4-hydroxy-tamoxifen (OHT) treatments on cell proliferation was analyzed using an MTS assay (CellTiter 96R AQueousOneSolution, Promega) and cells were counted with trypan blue in 96-wells flat bottom plates according to the manufacturer’s protocol. Cells were plated in the presence of estrogen or in estrogen-deficient medium for 24 h and increasing doses of OHT were added for 3 days. The relative percentage of living cells under these treatments was compared to medium-treated cells. To determine viable cells, three different experiments were performed in triplicates for each treatment. To obtain the relative percentage of viable cells, the number of live, medium treated cells was divided by the number of drug-treated cells.

### Statistical Analysis

For *in vitro* experiments statistical analyses were performed on at least 3 independent experiments using the student’s *t*-test or *ANOVA one-way analyses*. For *in vivo* mouse experiments, a two-sided Student *t* test was applied for comparison of continuous variables between animal groups. Differences were considered significant when the *p* values were<0.05.

Data from immunohistochemistry experiments were collected from five random fields of stained sections using Image Pro software version 4.5.0.29 (Media Cybernetics, Inc., Silver Spring, MD). Each field was quantified as a percentage of positively stained cells. Fields from the same tissue section were averaged and SPSS software version 12.0.2 (SPSS, Inc., Chicago, IL) was used to analyze the data.
